# Correlation o P16 and Bmi1 gene expression in human high grade glioma

**DOI:** 10.1186/1755-8166-7-S1-P9

**Published:** 2014-01-21

**Authors:** M K Sibin, GK Chetan, Ch Lavanya, Manoj M Jeru, Dhananjaya I Bhat

**Affiliations:** 1 Department of Human genetics, National Institute of Mental Health and neurosciences, Hosur road, Bangalore-29, India; 2 Department of Neurosurgery, National Institute of Mental Health and neurosciences, Hosur road, Bangalore-29, India

## Background

Gliomas are neoplasm of the central nervous system and make up approximately 80% of all malignant brain tumors. Even with the advancement in the field of surgery, chemotherapy and radiotherapy, the prognosis of glioma patients is dismal. Many molecular alterations of various oncogenes and tumor suppressor genes are seen in glioma and can be used for molecular characterization of tumor. The Bmi1 is a Polycomb group of protein which is associated with various cancers. The main alterations in Bmi1 gene in glioma includes copy number variation, over expression of mRNA and protein levels when compared to the non glioma brain samples. The CDKN2A (p16) is a tumor suppressor gene, mapped to 9p21. Alterations of the 9p21 locus have been implicated in many types of cancer, indicating the role of the tumor suppressor genes CDKN2A which encodes for p16^INK4a^/p14^ARF^. In this study we wanted to check the gene expression pattern of p16 and Bmi1 genes in glioma and their clinical correlations.

## Material and methods

50 glioma tissues were collected from Neurosurgery department and histologically confirmed as glioma. Total RNA was isolated and 1µg was converted to cDNA using RT kit. Real time RT PCR was performed using Taqman probes specific for p16 and Bmi1 and GAPDH as internal control.

## Results

We showed that the p16 mRNA levels in glioma found to be decreased when compared to the non glioma tissues. Bmi1 gene was found to be over expressed in glioma. There is a correlation in the expression of both genes. Bmi1 is the main upstream regulator of p16 and we found that gene expressions of both are correlated in glioma.

## Conclusions

It is concluded that p16 and Bmi1 genes are important in glioma and can be used as independent biomarker in glioma characterisation.

